# Deep Attention Fusion Hashing (DAFH) Model for Medical Image Retrieval

**DOI:** 10.3390/bioengineering11070673

**Published:** 2024-07-02

**Authors:** Gangao Wu, Enhui Jin, Yanling Sun, Bixia Tang, Wenming Zhao

**Affiliations:** 1 National Genomics Data Center, China National Center for Bioinformation, Beijing 100101, China; wugangao20@mails.ucas.edu.cn (G.W.); jineh@big.ac.cn (E.J.); sunyanling@big.ac.cn (Y.S.); 2 Beijing Institute of Genomics, Chinese Academy of Sciences, Beijing 100101, China; 3 University of Chinese Academy of Sciences, Beijing 100049, China; 4 CAS Key Laboratory of Genome Sciences and Information, Beijing Institute of Genomics, Chinese Academy of Sciences and China National Center for Bioinformation, Beijing 100101, China

**Keywords:** medical image retrieval, deep learning, deep hashing

## Abstract

In medical image retrieval, accurately retrieving relevant images significantly impacts clinical decision making and diagnostics. Traditional image-retrieval systems primarily rely on single-dimensional image data, while current deep-hashing methods are capable of learning complex feature representations. However, retrieval accuracy and efficiency are hindered by diverse modalities and limited sample sizes. Objective: To address this, we propose a novel deep learning-based hashing model, the Deep Attention Fusion Hashing (DAFH) model, which integrates advanced attention mechanisms with medical imaging data. Methods: The DAFH model enhances retrieval performance by integrating multi-modality medical imaging data and employing attention mechanisms to optimize the feature extraction process. Utilizing multimodal medical image data from the Cancer Imaging Archive (TCIA), this study constructed and trained a deep hashing network that achieves high-precision classification of various cancer types. Results: At hash code lengths of 16, 32, and 48 bits, the model respectively attained Mean Average Precision (MAP@10) values of 0.711, 0.754, and 0.762, highlighting the potential and advantage of the DAFH model in medical image retrieval. Conclusions: The DAFH model demonstrates significant improvements in the efficiency and accuracy of medical image retrieval, proving to be a valuable tool in clinical settings.

## 1. Introduction

Medical imaging, as an indispensable source of information for disease diagnosis and treatment planning, has experienced an explosive growth in data generated by various radiological imaging modalities in recent years. According to reports, there were 5 billion medical imaging studies conducted worldwide in 2009. By 2020, the volume of medical imaging data reached 35 zettabytes, representing a 44-fold increase from 2009 [1]. This surge has posed unprecedented challenges for the storage, management, and retrieval of medical imaging data. Continual advancements in pattern recognition and computer vision technology have significantly enhanced the role of medical imaging in assisting with disease diagnosis and assessment of diseases [2,3,4,5]. However, the high redundancy and variability of medical images present challenges to their interpretation.

Image retrieval aims to search for and obtain images from databases or online resources that are similar or related to an input image. Its primary objective is to find the most relevant matches from a large collection of images based on a given image or image description. Image-retrieval systems are widely used in various fields, including e-commerce, medical imaging, security surveillance, and social media [6].

Medical image retrieval, a specific domain of image retrieval, aims to identify the most pertinent matches amongst medical images. These matches can aid in disease diagnosis and assessment, spurring significant interest. Several methods have been developed for this task. Early conventional techniques relied heavily on manual text-annotation of images [7] or manual extraction of image features [8,9], whereas machine learning approaches suffered from the complexities inherent to feature engineering [10,11]. Furthermore, the efficiency of deep learning algorithms optimized for hashing [12,13] still remains influenced by the class information of the medical images, to some extent [14,15,16].

To address this problem, we collected multimodal medical image data of various cancer types from The Cancer Imaging Archive (TCIA) [17] and proposed the Deep Attention Fusion Hashing (DAFH) model for medical image retrieval. Our model employs a triplet network and self-attention mechanisms to optimize the extraction and fusion of multi-scale image features. Additionally, the introduction of focal loss enhances the model’s ability to recognize minority classes. Our experimental results demonstrate that our proposed model outperforms existing technologies in retrieval performance across different hash code lengths.

The main contributions of this paper are summarized as follows: we propose a novel deep hashing-based model for medical image retrieval, effectively processing medical images from diverse modality and types; we develop a triplet network model that integrates attention mechanisms and inter-layer features; and we utilize focal loss to enhance the recognition of minority samples, significantly improving retrieval performance on datasets. The structure of this paper is organized as follows: Section 2 summarizes the related works, Section 3 introduces the materials and methods, including data sources, data preprocessing, model architecture, and parameter details; Section 4 analyzes the experimental results and discusses the selection of model parameters; finally, Section 5 discusses the findings and limitations of the study, concludes the research, and explores its significance and potential applications in related fields.

## 2. Related Works

### 2.1. Conventional Medical Image Retrieval Algorithms

Early medical image retrieval primarily relied on manually extracted features from images, where experts annotated images with text descriptors to facilitate retrieval. This early method, known as Text-Based Image Retrieval (TBIR), primarily utilized textual data associated with images for search purposes [7]. TBIR was limited by the descriptive accuracy and the exhaustiveness of the text metadata, which often could not capture the full diagnostic detail presented in the images. As technology advanced, the field evolved into Content-Based Image Retrieval (CBIR), which represents a significant paradigm shift. CBIR systems analyze the visual content of the images themselves, rather than relying on external text descriptions [8,9]. These methods typically involved low-level features like texture, shape, and color, as well as algorithms such as Scale-Invariant Feature Transform (SIFT) to describe local features in images [8]. However, these manually extracted features face the “semantic gap” problem, making it difficult to express high-level abstract semantic information in images [18,19,20,21]. With the development of machine learning technology, traditional algorithms such as Support Vector Machines (SVM) and Random Forests began to be employed in medical image retrieval tasks [10,11]. These methods represented an improvement over manual feature extraction but were still limited by the complexity of feature engineering and their reliance on domain expertise.

### 2.2. Deep Hashing Algorithms

The introduction of hashing algorithms provided a new perspective for image retrieval. These algorithms map high-dimensional data into binary codes in a hash space and compute the distances between hash codes, to reduce storage and communication overheads. Image retrieval methods based on Locality-Sensitive Hashing (LSH) have been widely adopted for their efficiency [12,13]. In recent years, the rise of deep learning technology has revolutionized the field of medical image retrieval. Deep Convolutional Neural Networks (CNNs) can automatically learn complex feature representations from data, significantly improving retrieval accuracy [6,22,23]. With the continuous optimization of network architectures, such as AlexNet [24], VGG [25] and ResNet [26], the performance of medical image retrieval has been markedly enhanced.

Currently, deep hashing algorithms for medical image retrieval have been widely applied. For instance, Qayyum et al. [14] trained a two-stage medical image retrieval model using a multimodal dataset containing 24 categories across five modalities. However, this two-stage approach can lead to suboptimal results. To address this, Qiu et al. [15] utilized AlexNet to extract rich features from medical images and introduced a hashing layer composed of two fully connected layers before the output layer, thereby obtaining hash codes with multi-level semantic information through end-to-end feature fusion. Similarly, Fang et al. [16] designed an end-to-end model combining a triplet network with an attention mechanism within a residual network, achieving improved retrieval performance on the MIMIC-CXR and Fundus-iSee datasets. Nevertheless, the class information of medical images can still influence the retrieval results, to a certain extent.

## 3. Materials and Methods

Our study includes 913 samples from the TCIA database, totaling 535,948 medical images. We divided the training and retrieval data in 8:2, and all images underwent data cleaning and standardization. We developed a deep hashing method based on an attention-augmented triplet network for this experiment. Initially, we employed an EfficientNet feature extractor to capture the features of the images. Subsequently, we utilized an attention mechanism and inter-layer fusion to acquire multi-scale image features. Finally, by optimizing the model parameters through the triplet network, our model can output richly informative binary hash codes in an end-to-end manner.

### 3.1. Data Acquisition and Preprocessing

We collected medical images from 913 patients with ten different types of conditions from the TCIA database [27,28,29,30,31,32,33,34,35,36]. The specific categories of the collected medical images are detailed in Table 1. The patient samples included images of various cancer types, including but not limited to lung adenocarcinoma, esophageal cancer, and endometrial cancer, with all image samples acquired in DICOM format from the TCIA.

**Figure 1:**
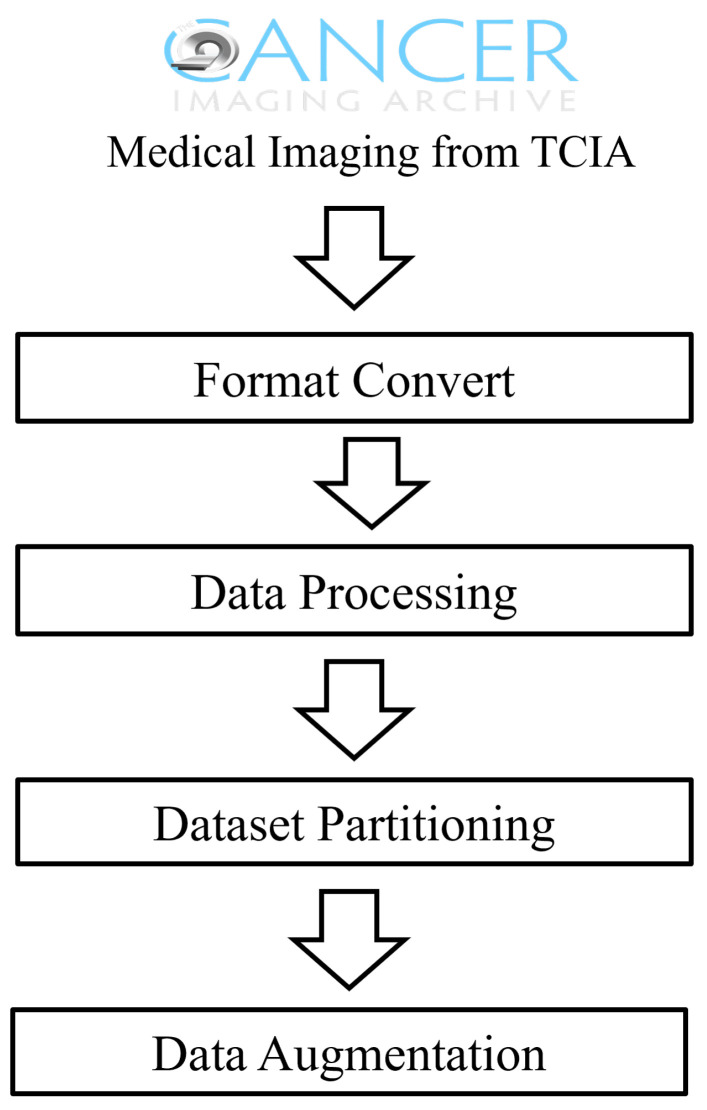
Flow chart for data preprocessing.

Figure 1 provides a detailed demonstration of the preprocessing. During the data preprocessing stage, we first converted DICOM images to a standardized PNG format to meet the input requirements of deep learning models. The image size was uniformly adjusted to 224 × 224 pixels to ensure data consistency. Data cleaning was a critical step in the preprocessing, where we computed the statistical parameters of the image data of mean, variance, and quartiles, and we used the Interquartile Range (IQR) method to identify and exclude outliers. After cleaning, a total of 372,004 radiographic image slices were retained in the TCIA dataset, providing a high-quality dataset for model training.

To enhance the model’s generalizability and simulate different imaging conditions, we implemented data augmentation on the image data, including adding noise, blurring, and brightness adjustments. Additionally, considering the multimodal nature of medical imaging data, we divided the dataset using an 8:2 stratified sampling method, ensuring diversity and balance in the training and test sets.

Ultimately, our preprocessing procedures provided a standardized, clean, and highly diverse medical imaging dataset for constructing a robust medical image retrieval system, offering a solid data foundation for achieving precise medical image retrieval.

### 3.2. Medical Image Retrieval Model Based on Deep Triplet Hashing Network

**Figure 2:**
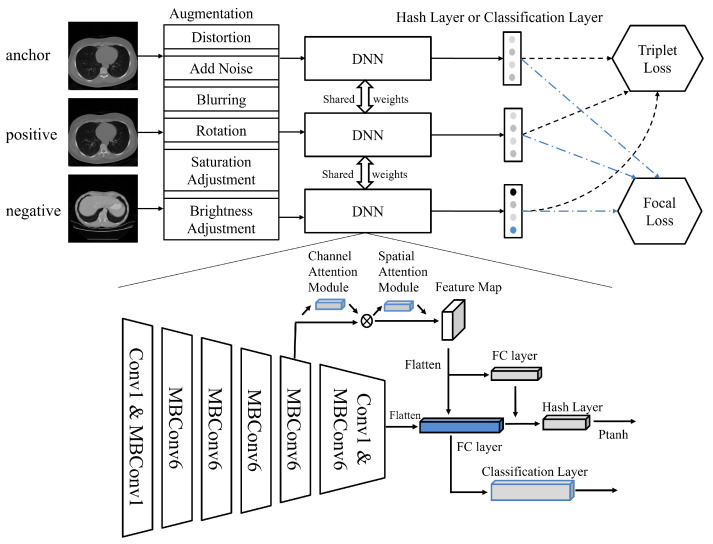
The architecture of DAFH.

Figure 2 provides an overview of the DHAF method used in this paper, which employs contrastive learning to integrate different types of data information from the same sample. The method described in this paper involves the following key stages:

- Data Augmentation: During the data preprocessing stage, we performed data augmentation on the collected medical image data, to enhance the model’s generalizability. This included operations such as rotation, scaling, and brightness adjustment to simulate different imaging conditions, thereby improving the model’s robustness against various transformations.
- Deep Feature Extraction: Utilizing EfficientNet from deep learning as the feature extractor, we independently extracted features from each medical image. EfficientNet is capable of capturing the deep features of images, providing rich information for subsequent image retrieval and classification tasks.
- Attention Mechanism and Inter-layer Feature Fusion: Building on the deep features, we introduced the Convolutional Block Attention Module (CBAM), to enhance the model’s focus on key areas within the images. Additionally, through inter-layer feature-fusion technology, we integrated deep features from different layers to obtain more comprehensive image representation.
- Binary Hash Code Retrieval: Finally, we mapped the continuous features to discrete binary hash codes, using a carefully designed quantization function. This process utilized a parameterized hyperbolic tangent function (Ptanh), which approximates the sign function, thus generating optimized hash codes for ease of similarity comparison during image retrieval.
  Through these four stages, the method proposed in this paper effectively extracts and integrates information from large-scale medical image data, achieving rapid and accurate medical image retrieval. The innovation of this method lies in its ability to handle multimodal data, as well as its adaptability to small samples and imbalanced datasets.

### 3.3. Architecture Details

#### 3.3.1. Feature Extraction Based on EfficientNet

EfficientNet [37] is an efficient network architecture that utilizes Neural Architecture Search (NAS) technology to optimize the network’s width, depth, and resolution. Through a Compound Scaling strategy, EfficientNet systematically scales the dimensions of various network layers, thereby achieving higher performance with limited resources. Furthermore, EfficientNet has shown superior performance in standard image recognition benchmarks, particularly excelling in complex medical imaging tasks, where it efficiently captures essential features crucial for enhancing the accuracy of medical image retrieval.

In this paper, we evaluated models such as EfficientNet and ResNet through a series of experimental comparisons. Ultimately, EfficientNet_b6 was selected. EfficientNet_b6 enables the model to capture more details and features. It incorporates advanced technologies including MBConv blocks (Mobile inverted Bottleneck Convolution) and SE blocks (Squeeze-and-Excitation blocks). These technologies enhance the model’s ability to recognize key features while maintaining parameter efficiency.

#### 3.3.2. Attention and Multi-Layer Feature Fusion

In this study, we adopted EfficientNet as the backbone network and integrated a Convolutional Block Attention Module (CBAM) [38] to enhance the expressive power of the features. We selected the output of the penultimate Modified Block Convolution (MBConv) module from EfficientNet as the input for the CBAM, based on the premise that deeper network layers contain rich spatial hierarchical information, which is ideal for further attention mechanism processing. The CBAM module adaptively adjusts the weights of the feature map by introducing channel and spatial attention mechanisms, thereby enhancing the expressive capacity of the features. Specifically, the Channel Attention Module (CAM) selectively enhances features across channels based on their informational importance. The Spatial Attention Module (SAM) further emphasizes significant spatial features, guiding the network’s focus within the image.

The output of CBAM is concatenated with the output from EfficientNet, integrating features from different processing stages and enhancing the model’s ability to capture both global and local features. Specifically, the output from CBAM, denoted as $F_{\mathrm{CBAM}}$, is concatenated with the output from EfficientNet, $F_{\mathrm{EfficientNet}}$, to form a unified feature representation $F_{\mathrm{Concat}}$ through the following operation:

(1) $$F_{\mathrm{Concat}}=\mathrm{Concat}\left(F_{\mathrm{CBAM}},F_{\mathrm{EfficientNet}}\right)$$

The concatenated features $F_{\mathrm{Concat}}$ are then subjected to further feature transformation through a linear layer ${\mathrm{Linear}}_{1}$:

(2) $$F_{\mathrm{Linear}1}=W_{L1}\cdot F_{\mathrm{Concat}}+b_{L1}$$

Here, $W_{L1}$ and $b_{L1}$ represent the weight matrix and bias term of the ${\mathrm{Linear}}_{1}$ layer, respectively.

The output from the linear layer $F_{\mathrm{Linear}1}$ is bifurcated into two parts: one part is directed to the classification layer for image categorization tasks, where a softmax function is used to output class probabilities; the other part is concatenated with the flattened CBAM output $F_{\mathrm{CBAM}}$ and then processed through another linear layer ${\mathrm{Linear}}_{2}$:

(3) $$F_{\mathrm{Flatten}}=\mathrm{Flatten}\left(F_{\mathrm{CBAM}}\right)$$

(4) $$F_{\mathrm{ConcatHash}}=\mathrm{Concat}\left(F_{\mathrm{Linear}1},F_{\mathrm{Flatten}}\right)$$

(5) $$F_{\mathrm{Linear}2}=W_{L2}\cdot F_{\mathrm{ConcatHash}}+b_{L2}$$

Ultimately, $F_{\mathrm{Linear}2}$ is fed into the hashing layer to produce hash codes, where $W_{L2}$ and $b_{L2}$ represent the weight matrix and bias term of the ${\mathrm{Linear}}_{2}$ layer, respectively.

This design enables our model not only to perform effective image classification but also to generate hash codes for image retrieval, greatly enhancing the practicality and efficiency of the model in medical image analysis.

#### 3.3.3. Learnable Quantization Hashing Layer

To effectively conduct image retrieval, this study introduced a hash code layer at the final output stage of the model. Image retrieval tasks require the model to map learned features into a discrete hash space to facilitate rapid comparison and retrieval. To this end, we designed a hash code layer that utilized learnable parameters to optimize the binarization of feature vectors.

The core of the hash code layer is a learnable hyperbolic tangent function tanh(·), whose parameter α can be adjusted during the model training process. This function aims to map continuous features into a more compact representation space while maintaining the discriminative ability between features. Specifically, the features processed by the linear layer ${\mathrm{Linear}}_{2}$, denoted as $F_{\mathrm{Linear}2}$, are quantized through the hyperbolic tangent function to generate the final hash code *H*:

(6) $$H=\tanh(\alpha \cdot F_{\mathrm{Linear}2}+b_{L2})$$

where α is a learnable parameter, and we require α>1.

This design not only improves the efficiency of image retrieval but also enhances the model’s sensitivity to feature similarity, enabling the generation of more compact and discriminative hash codes. This is of significant importance for rapid retrieval and comparison in large-scale image databases.

#### 3.3.4. Classification and Triplet Loss

In our study, to train an efficient medical image retrieval model, we adopted a multitask learning strategy that combines classification loss and triplet loss. Below is a detailed description of the two key loss functions involved in constructing the objective function.

The classification loss is based on focal loss [39], a loss function designed specifically to address class imbalance issues and improve the model’s ability to recognize minority classes. We utilize the output of the classification layer to compute focal loss, defined as follows:

(7) $$L_{focal}\left(p_{t}\right)=-{\left(1-p_{t}\right)}^{\gamma }\log\left(p_{t}\right)$$

Here, $p_{t}$ represents the probability predicted by the model that the sample belongs to the positive class, and γ is a tuning parameter used to balance the importance of easy-to-classify and hard-to-classify samples.

For multi-class problems, focal loss can be extended to the weighted sum of all classes:

(8) $$L_{focal}\left(x\right)=-\sum_{i=1}^{n}\left[y=i\right]{\left(1-p_{i}\right)}^{\gamma }\log\left(p_{i}\right)$$

Here, *n* is the total number of classes, and [y=i] is the indicator function, which equals 1 if the sample’s true label is *i*, and 0 otherwise.

Triplet loss [40] is used to learn discriminative representations of image features, ensuring that images of the same class have smaller feature distances compared to images of different classes. In our study, we simultaneously input anchor, positive, and negative samples into feature extractors with the same parameters, where positive samples represented data of the same class as the anchor, and negative samples represented data of different classes. The calculation of triplet loss is as follows:

(9) $$L_{triplet}=\sum_{i=1}^{N}\max\left(0,d\left(a_{i},p_{i}\right)-d\left(a_{i},n_{i}\right)+margin\right)$$

Here, $a_{i}$, $p_{i}$, and $n_{i}$ represent the feature representations of anchor, positive, and negative samples, respectively, d(·,·) is the distance metric between features, and margin is the minimum distance maintained between positive and negative sample pairs.

Combining the two loss functions mentioned above, we construct the objective function as follows:

(10) $$L_{total}=\lambda \left(L_{focal}+L_{focal_{P}}+L_{focal_{N}}\right)+\beta L_{triplet}$$

In this objective function, $L_{focal}$, $L_{focal_{P}}$, and $L_{focal_{N}}$ represent the Focal Losses of anchor, positive, and negative samples, respectively, while $L_{triplet}$ represents the triplet loss; λ and β are hyperparameters used to balance the classification loss and triplet loss weights, with λ set to 1/3 in our experiments. During model training, we minimize the objective function $L_{total}$ to simultaneously optimize classification accuracy and feature discriminability, thereby improving the performance of medical image retrieval.

#### 3.3.5. Training and Implementation Details

In this study, we employed the PyTorch deep learning framework for model construction and training. Model training was conducted on a high-performance computing platform equipped with Nvidia V100 32G GPUs, to ensure computational efficiency. The data were partitioned into a database set (training set) and a test set in an 8:2 ratio.

Table 2 shows the parameter settings we used during the model training. During training, we utilized the Adam optimizer with a learning rate set to 1 × $10^{-4}$. The training consisted of 50 epochs, with the margin hyperparameter for triplet loss set to 0.5 and the gamma hyperparameter for focal loss set to 1.5. The objective of the training was to minimize the total loss function $L_{total}$, which integrates classification loss and triplet loss, as detailed in Algorithm 1.
**Algorithm 1** Deep Hashing Network Training1:**Initialize:**2:Set model parameters: epochs = 50, learning_rate = 1 × $10^{-4}$3:optimizer = Adam, loss_function = triplet loss + focal loss4:**Load Data:**5:Load dataset from TCIA6:Preprocess and augment data7:Split data into training and testing sets8:**Build Model:**9:Use EfficientNet as the base feature extractor10:Add an attention mechanism module (e.g., CBAM)11:Integrate features into deep hashing layer12:Configure outputs for classification and hashing layers13:**Training Loop:**14:**for** epoch = 1 to epochs **do**15:  **for** data, labels in training_data **do**16:    optimizer.zero_grad()17:    features = EfficientNet(data)18:    attention_features = CBAM(features)19:    hash_codes = DeepHashLayer(attention_features)20:    classification_output = ClassifierLayer(hash_codes)21:    triplet_loss = compute_triplet_loss(hash_codes, labels)22:    focal_loss = compute_focal_loss(classification_output, labels)23:    total_loss = triplet_loss + focal_loss24:    total_loss.backward()25:    optimizer.step()26:  **end for**27:  Validate model on test data28:**end for**29:**Save Model:**30:Save trained model parameters to file31:**Output:**32:Print loss and validation results during training

#### 3.3.6. Metrics

In our study, the core metric for evaluating retrieval performance is Mean Average Precision (MAP), which assesses overall performance across the dataset. Compared to traditional precision and recall metrics, MAP offers superior performance and stability.

Average Precision (AP) quantifies the average proportion of correct results among all returned results for a single query image. It is calculated using the following formula:

(11) $$\mathrm{AP}\left(q\right)=\frac{1}{n_{\mathrm{gt}}}\sum_{k=1}^{n}P\left(k\right)\times r\left(k\right)$$

Here, $n_{\mathrm{gt}}$ represents the total number of correct results in query image *q*, *n* is the total number of returned results, P(k) denotes the precision at rank *k*, and r(k) is an indicator function that takes a value of 1 when the result at rank *k* is correct, and 0 otherwise.

MAP is computed by averaging the AP values for all query images. Assuming there are *q* query images, the formula for MAP is

(12) $$\mathrm{MAP}=\frac{1}{\left|Q\right|}\sum_{q=1}^{\left|Q\right|}\mathrm{AP}\left(q\right)$$

Additionally, the MAP@k metric is used to measure average precision when considering the top *k* retrieval results. Specifically, when *k* is equal to 1, MAP@1 considers only the first result in the retrieval, reflecting the accuracy of the system’s first returned result. In tasks such as information retrieval, MAP@1 can serve as an indicator of classification accuracy.

## 4. Results

To enhance the quality of model retrieval, we believe it is necessary to improve the model’s discriminative capability for images. To this end, we employed focal loss to assist in enhancing the ranking quality of the model. Additionally, to capture information from few-shot samples, we utilized triplet loss to improve the model’s recognition capability for these samples. Furthermore, to better leverage information from regions of interest and multiple scales, we incorporated inter-layer feature fusion to fully exploit multi-scale image information.

### 4.1. Comparative Analysis of Retrieval Performance

As shown in the Table 3, we compared our model’s performance on the TCIA dataset with current advanced models. Specifically, we compared our approach with advanced image retrieval models such as HashNet [41], DPSH [42], DSH [43], and DPN [44]. A higher Mean Average Precision (MAP) indicates that more images of the same category as the query image were retrieved. Thus, we calculated the MAP@10 for each model under hash code lengths of 16-bit, 32-bit, and 48-bit. Our method achieved impressive performances of 0.711, 0.754, and 0.762 in MAP@10, respectively. In contrast, other advanced methods like DSH, HashNet, DPSH, and DPN showed relatively lower performance on the same dataset, indicating that our proposed method effectively addresses the ranking problem.

**Figure 3:**
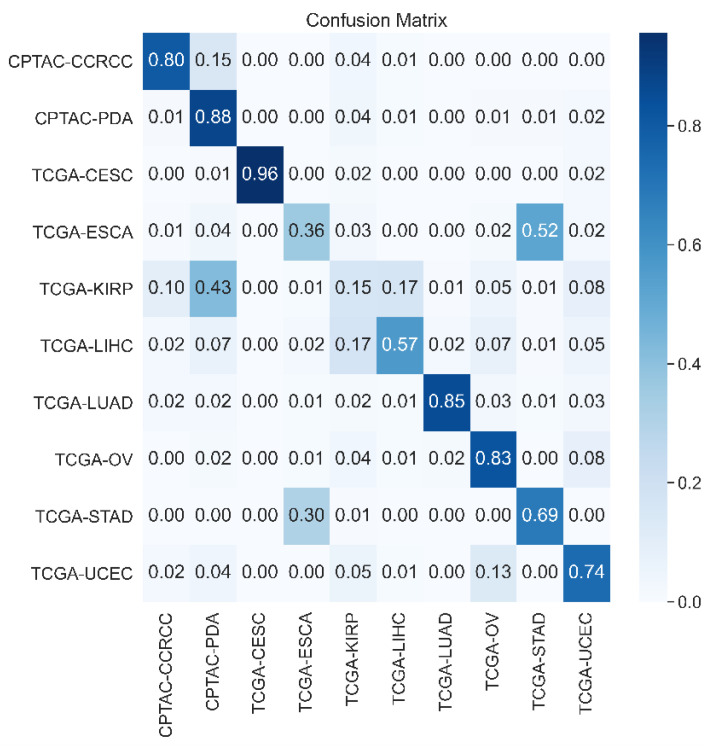
The confusion matrix of MAP@1.

Additionally, MAP@1 is a crucial metric for evaluating the accuracy of the first retrieval result. In the context of medical image retrieval, quickly and accurately providing the most relevant images is vital for assisting doctors in diagnosis. As shown in Figure 3, our experiments revealed that the proposed model achieved a MAP@1 of 0.745 with a 32-bit hash code length. This result indicated that the model could rank the most relevant image first in over 74.5% of the cases. Despite this high efficiency, we conducted a confusion matrix analysis, as shown in the figure. The analysis revealed that the model had relatively lower MAP@1 values when dealing with specific types of medical images, such as TCGA-KIRP, TCGA-LIHC, and TCGA-ESCA. This may be due to the high visual similarity of these image types, posing a challenge for the model in distinguishing between them.

### 4.2. Impact of Backbone Network Architecture on Results

To validate the impact of different backbone networks on model performance, we tested the retrieval effectiveness of our model using various backbone networks. These networks included the traditional ResNet-50, ResNet-50 with Attention, a combination of ViT [45] and ResNet for feature fusion, and the EfficientNet architecture. The experimental results are summarized in the table.

As shown in Table 4, when using ResNet-50 as the backbone network, the model achieved a MAP@10 of 0.658 with a 32-bit hash code length. Introducing an attention mechanism into ResNet-50 improved the model’s performance to a MAP@10 of 0.685 with the same hash code length. This demonstrates that the attention mechanism enhances the model’s ability to capture key features, thereby improving retrieval performance.

The method combining ViT and ResNet features achieved a MAP@10 of 0.670 with a 16-bit hash code length and improved to 0.685 with a 32-bit hash code length. These results indicate that fusing features from different architectures can provide complementary information and improve the model’s generalization capability, though the improvement is limited.

Finally, using EfficientNet as the backbone network resulted in a MAP@10 of 0.711 with a 16-bit hash code length, further improving to 0.754 with a 32-bit hash code length. The superior performance of EfficientNet is attributed to its compound scaling method, which optimizes the network structure by simultaneously scaling depth, width, and resolution.

The experimental results demonstrate that different backbone networks significantly affect the model’s retrieval performance. EfficientNet, due to its advanced network design, performed the best among the tested architectures. The attention mechanism and feature-fusion strategies also proved effective in enhancing retrieval accuracy. Therefore, we ultimately chose EfficientNet as the backbone network.

### 4.3. Ablation Analysis of Our Model

Finally, to quantify the specific contributions of various components to the model’s performance, we conducted ablation studies. Specifically, we focused on three key modules: the attention mechanism (AT), inter-layer feature fusion (LF), and the learnable quantization function (Ptahn).

As shown in Table 5, adding individual components, such as Ptanh, the attention mechanism, and the inter-layer fusion module, to the baseline model increased the model’s performance. Notably, the inter-layer fusion module and the attention mechanism provided significant performance improvements, boosting the MAP@10 value by approximately 25% compared to the original EfficientNet model. When comparing different component combinations, the model incorporating both Ptanh and the attention mechanism showed a slight performance improvement, whereas other combinations resulted in slight performance decreases. Ultimately, the proposed model achieved the best performance when utilizing all the components together.

The ablation studies demonstrated that the proposed model, DAFH, achieved the highest performance, indicating that the inclusion of these modules effectively enhances performance. These results affirm that each component in our model plays a positive role in improving retrieval performance, further validating the efficacy of our approach.

## 5. Conclusions

This study aimed to develop a deep hashing-based medical image retrieval technique to enhance the efficiency and accuracy of medical image retrieval for clinicians. Our proposed model underwent extensive testing on the TCIA dataset that we constructed, and it was compared with existing medical image retrieval methods. The results demonstrate that the DAFH model achieved all higher retrieval performances under hash code lengths of 16-bit (mAP@10 of 0.711), 32-bit (mAP@10 of 0.754), and 48-bit (mAP@10 of 0.762) when evaluated by MAP@10. And the DAFH model achieved MAP@1 of 0.745 with a 32-bit hash code length. The designed module and the methods used in the DAFH contributed to the high performance. The CBAM module, by adaptively adjusting the channel and spatial weights of the feature maps, enabled the model to focus more on the diagnostically valuable parts of the images. The inter-layer feature-fusion strategy enhanced the model’s ability to capture both global and local information in medical images by integrating features from different depth layers. The use of a learnable tanh function as the quantization layer allowed the model to better adapt to various data distributions and retrieval tasks.

Despite the positive outcomes of this study, several limitations exist. Firstly, the model’s performance is somewhat influenced by the size and quality of the dataset. Secondly, for rare disease types, the model’s performance may be limited by the diversity and representativeness of the training data. Additionally, computational complexity and real-time performance are issues that need to be addressed in future work. We have also recognized potential limitations in our data preprocessing, where standardization based on mean and variance might exclude important data points, potentially introducing selection bias. This highlights the need for future research to investigate excluded data and explore more inclusive methodologies, such as the application of large models and 3D-based models, to enhance the robustness and credibility of our findings.

Future research could explore several directions. Firstly, we will continue to monitor and update the DAFH model in three aspects: (1) We will increase the variety and quantity of the medical imaging data, to improve the model’s generalization ability and robustness. (2) We will investigate more efficient attention mechanisms and feature-fusion strategies to further enhance model performance. (3) We will explore the potential of large language models, which have demonstrated superior data representation capabilities in various fields. Although the improvement of large language models in medical imaging tasks is not yet significant, this might be due to the untapped potential of these models. Considering their successful application in multiple domains in recent years, future research could explore the effectiveness of large language models in medical image retrieval. Secondly, to facilitate the use of Deep Attention Fusion Hashing (DAFH), we plan to employ various strategies, such as developing and releasing training modules on GitHub and establishing an email group accompanied by discussion workshops to garner feedback and suggestions. At present, DAFH has been integrated into OBIA [46] (https://ngdc.cncb.ac.cn/obia/home (accessed on 10 June 2024)) for medical image retrieval, and further integration into other existing clinical workflows needs further exploration, considering factors such as real-time processing demands, system compatibility, and explainability. In our future work, we intend to incorporate interpretability techniques to enhance the model’s explainability, aiming to build user trust and improve the effectiveness of clinical applications. Moreover, we advocate for a holistic interdisciplinary collaboration to advance DAFH research. This would have clinicians validate the image retrieval results using their domain knowledge, data scientists annotate and outline the medical image to improve retrieval accuracy, and ethicists emphasize the standardized use of medical images.

## Figures and Tables

**Table 1:** Data type from TCIA.

Collection	Cancer Type	Location	Subjects	Data Type
TCGA-KIRP	Kidney Renal Papillary Cell Carcinoma	Kidney	33	CT, MR, PT, Pathology
TCGA-LIHC	Liver Hepatocellular Carcinoma	Liver	97	MR, CT, PT, Pathology
CPTAC-CCRCC	Clear Cell Carcinoma	Kidney	222	Clinical, Genomics, Proteomics
TCGA-UCEC	Uterine Corpus Endometrial Carcinoma	Uterus	65	CT, CR, MR, PT, Pathology
TCGA-OV	Ovarian Serous Cystadenocarcinoma	Ovary	143	CT, MR, Pathology
TCGA-ESCA	Esophageal Carcinoma	Esophagus	16	CT, Pathology
CPTAC-PDA	Ductal Adenocarcinoma	Pancreas	168	CT, MR, PT, US, Pathology
TCGA-STAD	Stomach Adenocarcinoma	Stomach	46	CT, Pathology
TCGA-CESC	Cervical Squamous Cell Carcinoma and Endocervical Adenocarcinoma	Cervix	54	MR, Pathology
TCGA-LUAD	Lung Adenocarcinoma	Lung	69	CT, PT, NM, Pathology

**Table 2:** Parameters Settings.

Parameters	Value
Epochs	50
Learning Rates	1 × $10^{-4}$
Optimizer	Adam
Regularization Rate	1 × $10^{-4}$
Triplet Loss Margin	0.5
Focal Loss Gamma	1.5

**Table 3:** The performances of different methods.

Model	16-bit	32-bit	48-bit
HashNet	0.550	0.727	0.736
DSH	0.578	0.581	0.596
DPSH	0.540	0.717	0.740
DPN	0.679	0.670	0.681
Ours (DAFH)	**0.711**	**0.754**	**0.762**

**Table 4:** Comparison of different backbone neural networks.

Model	16-bit	32-bit	48-bit
ResNet-50	-	0.658	-
ResNet-50+Attention	0.584	0.685	0.705
ViT+ResNet-50	0.670	0.685	0.691
EfficientNet	**0.711**	**0.754**	**0.762**

**Table 5:** Ablation analysis of different modules.

Model	MAP@1	MAP@10
EfficientNet	0.370	0.498
EfficientNet+Ptanh	0.405	0.532
EfficientNet+AT	0.718	0.734
EfficientNet+LF	0.716	0.742
EfficientNet+Ptanh+AT	0.653	0.695
EfficientNet+Ptanh+LF	0.704	0.705
EfficientNet+AT+LF	0.691	0.728
Proposed	**0.745**	**0.754**

## Data Availability

All imaging data utilized in the present study are publicly accessible through TCGA-KIRP (https://www.cancerimagingarchive.net/collection/tcga-kirp/ (accessed on 10 June 2024)), TCGA-LIHC (https://www.cancerimagingarchive.net/collection/tcga-lihc/ (accessed on 10 June 2024)), CPTAC-CCRCC (https://www.cancerimagingarchive.net/collection/cptac-ccrcc/ (accessed on 10 June 2024)), TCGA-UCEC (https://www.cancerimagingarchive.net/collection/tcga-ucec/ (accessed on 10 June 2024)), TCGA-OV (https://www.cancerimagingarchive.net/collection/tcga-ov/ (accessed on 10 June 2024)), TCGA-ESCA (https://www.cancerimagingarchive.net/collection/tcga-esca/ (accessed on 10 June 2024)), CPTAC-PDA (https://www.cancerimagingarchive.net/collection/cptac-pda/ (accessed on 10 June 2024)), TCGA-STAD (https://www.cancerimagingarchive.net/collection/tcga-stad/ (accessed on 10 June 2024)), TCGA-CESC (https://www.cancerimagingarchive.net/collection/tcga-cesc/ (accessed on 10 June 2024)), TCGA-LUAD (https://www.cancerimagingarchive.net/collection/tcga-luad/ (accessed on 10 June 2024)).
